# Chlorhexidine versus povidone-iodine for surgical site infection prevention: an updated meta-analysis and trial sequential analysis of randomized controlled trials

**DOI:** 10.3389/fmed.2025.1641815

**Published:** 2025-11-20

**Authors:** Shengyi Yang, Zhenwei Li, Feiyu Wu, Liyuan Sun, Yulu He, Changxian Wang

**Affiliations:** Department of Infection Control, Second Affiliated Hospital of Zhejiang University School of Medicine, Hangzhou, Zhejiang, China

**Keywords:** chlorhexidine, povidone-iodine, surgical site infection, meta-analysis, randomized controlled trials

## Abstract

**Background:**

Chlorhexidine (CHX) and povidone-iodine (PVI) are the most commonly used antiseptic agents for preoperative skin preparation to prevent surgical site infections (SSIs). This meta-analysis aimed to determine the superior agent between them for SSI prevention.

**Methods:**

We conducted a systematic review and meta-analysis in accordance with the Preferred Reporting Items for Systematic Reviews and Meta-Analyses (PRISMA) guidelines. A comprehensive search of electronic databases (PubMed, Web of Science, Embase, and Cochrane Central Register of Controlled Trials) was performed from inception to 1 May 2025, to identify relevant randomized controlled trials (RCTs). Heterogeneity was assessed using the chi-squared (Q) test and the I^2^ statistic. A random-effects model was applied when significant heterogeneity was present. The robustness of the findings was evaluated using trial sequential analysis (TSA) with a random-effects model. All statistical analyses were performed using Review Manager.

**Results:**

A total of 32 high-quality RCTs, involving 29,748 participants, were included. The pooled analysis using a random-effects model demonstrated that CHX was significantly more effective than PVI in preventing SSIs (RR = 0.83, 95% CI 0.72–0.95, *p* = 0.009). Subgroup analysis by wound classification revealed that CHX was superior to PVI in clean-contaminated surgeries (11 RCTs; RR = 0.75, 95% CI 0.62–0.92, *p* = 0.004), but no significant difference was observed in clean surgeries (20 RCTs; RR = 0.90, 95% CI 0.67–1.20, *p* = 0.46). Further stratification by SSI type indicated that CHX significantly reduced the risk of superficial incisional SSIs (18 RCTs; RR = 0.82, 95% CI 0.69–0.98, *p* = 0.03), but not deep incisional SSIs (16 RCTs; RR = 0.95, 95% CI 0.76–1.18, *p* = 0.63) or organ-space SSIs (11 RCTs; RR = 1.13, 95% CI 0.89–1.42, *p* = 0.32). Additionally, CHX was associated with a significantly lower risk of bacterial decolonization (RR = 0.38, 95% CI 0.26–0.57, *p* < 0.001) and febrile episodes (RR = 0.57, 95% CI 0.35–0.92, *p* = 0.02) compared to PVI. The TSA confirmed the robustness of these findings, indicating that the cumulative evidence was sufficient and conclusive.

**Conclusion:**

CHX-based antiseptics are more effective than PVI-based ones in preventing overall SSIs, particularly in clean-contaminated procedures. The superiority of CHX is primarily evident in reducing superficial incisional SSIs, with no significant advantage observed for deep incisional or organ-space SSIs.

## Introduction

Surgical site infections (SSIs) are among the most common healthcare-associated infections (HAIs). Approximately 11% of patients undergoing general surgery will develop surgical incision sites 30 days after surgery (1). They are associated with longer post-operative hospital stays, additional surgical procedures, and higher mortality (2). The World Health Organization (WHO) suggests that preoperative skin antisepsis is one of the most critical factors for postoperative SSIs (3).

Povidone iodine (PVI) is a preeminent antiseptic measure in surgery that does not induce resistance or cross-resistance to antibiotics and is economically reasonable. Chlorhexidine (CHX), a broad-spectrum antimicrobial agent that damages bacterial cytoplasmic membranes without causing bacterial resistance, is a possible alternative antiseptic agent (4). Some clinical practice guidelines recommend the use of antiseptic skin solutions containing CHX gluconate and iodophor to prevent SSIs; however, there is a lack of consensus regarding the most effective agent (3, 5). A meta-analysis of high-quality randomized controlled trials (RCTs) did not show any benefit of alcoholic chlorhexidine skin preparation, which is more expensive than other readily available alternatives (6). However, some other meta-analyses revealed that chlorhexidine was superior to PVI in preventing postoperative SSIs (7, 8). Furthermore, some high-quality RCTs have been implemented recently, providing strong evidence of controversy regarding the most effective agent.

Considering the contradiction of the results and the publication of large, randomized trials in recent years, this justifies the need for updated meta-analyses. This meta-analysis aimed to evaluate the effects of chlorhexidine and PVI on SSI prevention.

## Materials and methods

This systematic review and meta-analysis adhered to the Preferred Reporting Items for Systematic Reviews and Meta-analysis (PRISMA) guidelines (9).

### Search strategy

A systematic literature search was conducted by two investigators (SY and ZL) in collaboration with an experienced medical librarian (LW). We searched the electronic databases of PubMed, Web of Science, Embase, and the Cochrane Central Register of Controlled Trials from their inception to 1 May 2025. The search strategy used a combination of keywords and subject headings related to “chlorhexidine,” “povidone-iodine,” and “randomized controlled trial.” The full search syntax for all databases is provided in Supplementary Table 1. The search was restricted to English-language publications. To ensure comprehensive coverage, we also manually screened the reference lists of all included studies and relevant review articles. All identified records were imported into EndNote X9 (Clarivate Analytics), and duplicates were removed electronically.

### Inclusion criteria and exclusion criteria

Two investigators independently screened the titles and abstracts of all identified records for eligibility. Subsequently, the full texts of potentially relevant studies were retrieved and assessed independently against the predefined inclusion criteria. Any discrepancies between the reviewers were resolved through consensus or through consultation with a third investigator (FW). The inclusion criteria were as follows:

Patients: Preoperative skin antisepsis in adult patients (>18y);
Intervention: The CHX-containing solution was reoperative skin antiseptic around the surgical site, whether alcoholic or aqueous.
Control: A PVI-containing solution was used for skin disinfection around the surgical site, whether alcoholic or aqueous.
Outcomes: Reported outcomes of interest, total SSIs, superficial incisional infection, deep incisional infection, and organ space infection.
Study design: All RCTs.

The exclusion criteria were: (1) non-surgical or pediatric (<18 years) populations, or patients with chlorhexidine/povidone-iodine allergy; (2) use of non-comparator antiseptics or combination regimens; (3) absence of a control group, animal/*in vitro* studies, or insufficient outcome data for RR calculation; (4) studies on indwelling catheters or blood sampling procedures; and (5) gray literature, including conference abstracts, reviews, editorials, and case reports.

### Data extraction

Data were systematically extracted from the included studies using a standardized form. The extracted information included: first author, publication year, participant sex and age, sample size, surgical type, wound classification, follow-up duration, details of the intervention and comparator groups, and primary/secondary outcomes. When multiple similar studies were published by the same author or group, only the most recent publication was included to avoid data overlap. For trials with more than two intervention arms, only data relevant to the chlorhexidine and povidone–iodine groups were extracted. The primary outcome was the incidence of postoperative surgical site infections (SSIs). Secondary outcomes included bacterial decolonization and episodes of fever. When critical data were missing or unclear, the corresponding authors were contacted for clarification.

### Quality assessment

The quality of all RCTs was assessed using the Cochrane evaluation criteria, which included the following domains: random sequence generation, allocation concealment, performance bias, detection bias, attrition bias, reporting bias, and other sources of bias. There are three levels of bias: low, unclear, or high (10).

### Data synthesis and statistical analysis

A meta-analysis was performed to pool the relative risk of each study. Chi-squared-based Q test and I^2^ were used to evaluate the heterogeneity within the studies. The random-effects meta-analysis model was used when the heterogeneity was statistically significant (*I*^2^ > 50%, *p* < 0.05) (11). Significant heterogeneity was assessed using subgroup, sensitivity, and descriptive analyses. Leave-one-out sensitivity analyses were performed by removing a single study each time to assess whether the results of this meta-analysis were robust. Publication bias was explored using the funnel plot method by graphing the effect size of the trials on the horizontal axis and the number of participants in each trial on the vertical axis. Asymmetry in the funnel plot suggested publication bias.

Trial sequential analysis (TSA) was performed to evaluate the statistical power of the current sample size using TSA software (12).^1^ The heterogeneity-adjusted required information size was calculated using a two-sided conventional boundary with a 5% type I error rate and an 80% statistical power.

The meta-analysis was conducted using Review Manager (v5.3), and leave-one-out analysis was conducted using STATA software (v15.0, College Station, TX, United States).

TSA was conducted with TSA 0.9.5.10 Beta software (Copenhagen Trial Unit, Centre for Clinical Intervention Research, Copenhagen).

## Results

### Identification of studies

**Figure 1:**
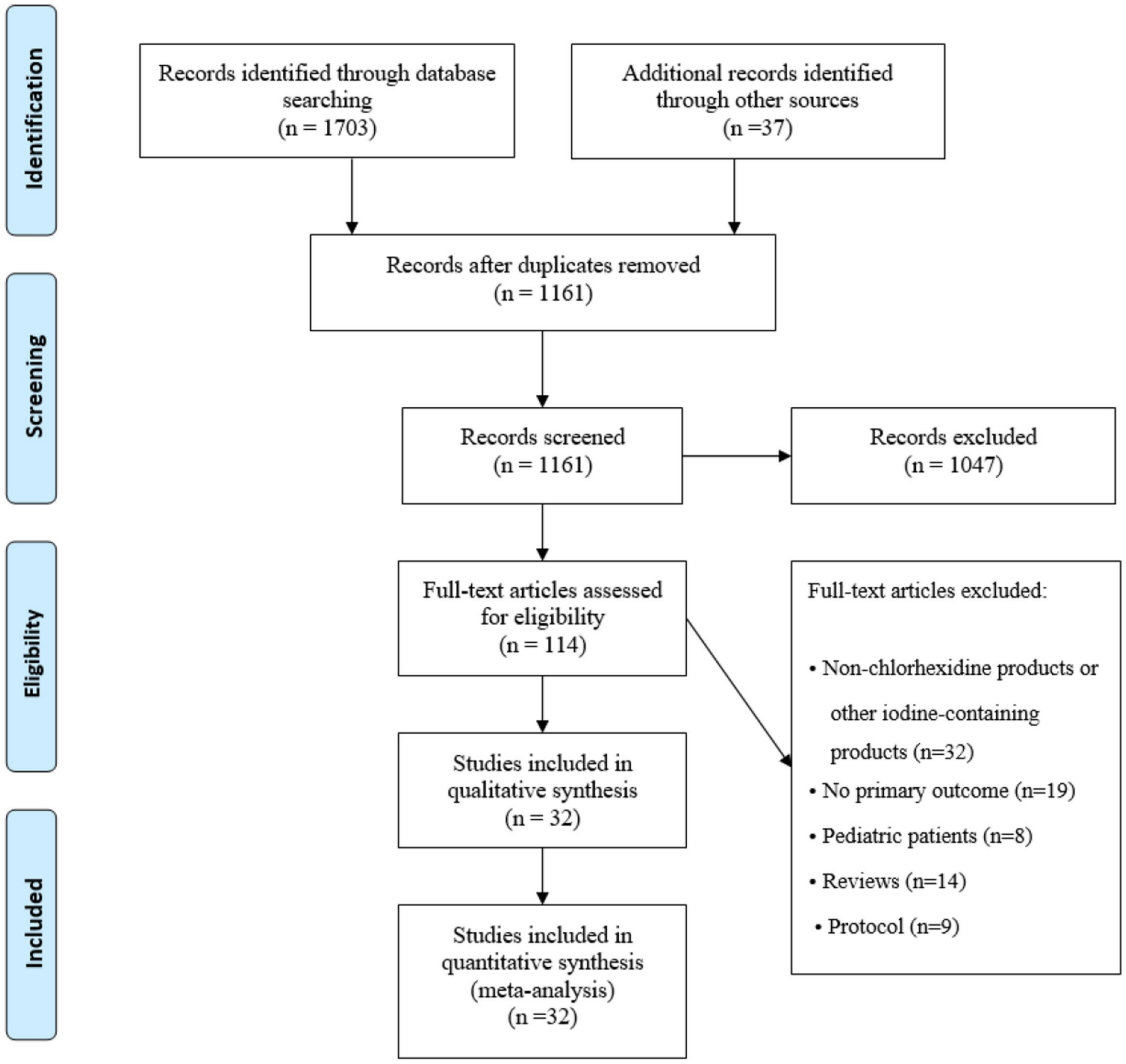
Flow diagram of the study selection process.

The search strategy yielded a total of 1703 abstracts from four English databases, while a manual search of the references cited in other available articles and previous reviews yielded an additional 37 abstracts. After removing duplicates and screening the abstracts, 114 studies were included. After the full-text articles were assessed for eligibility, 82 studies were excluded: 32 studies were for non-chlorhexidine products or other iodine-containing products, 19 studies had no primary outcome, 8 studies were for pediatric patients, 14 studies were for reviews, and 9 studies were for protocols. Finally, 32 studies were included in the quantitative synthesis (Figure 1).

### Characteristics of the involved studies

**Figure 2:**
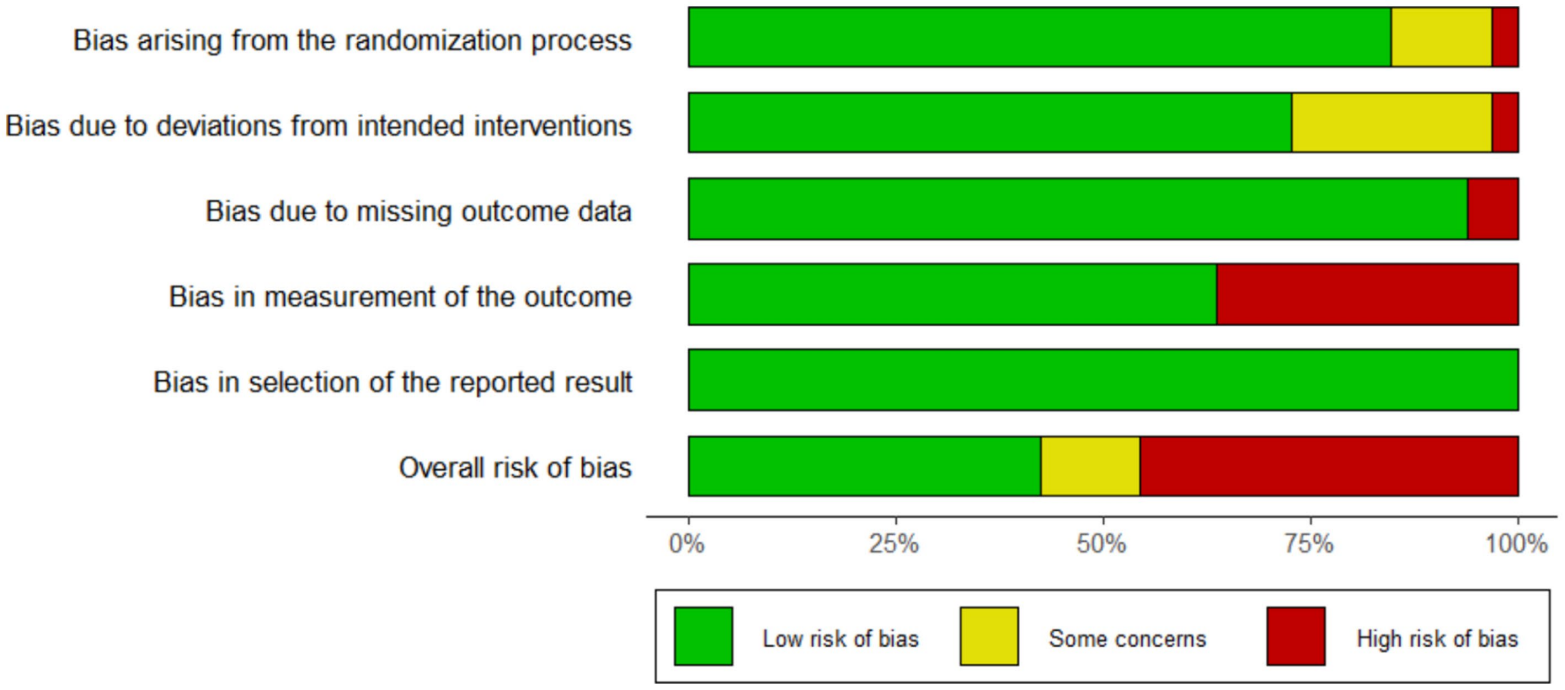
Risk of bias graph: review authors’ judgments about each risk of bias item presented as percentages across all included studies.

The baseline characteristics of the 32 included studies, comprising a total of 29,748 participants, are summarized in Table 1. Of these, 14,473 (48.6%) patients were preoperatively prepared with chlorhexidine (CHX), while 15,275 (51.4%) received povidone-iodine (PVI). According to wound classification, 8 studies involved clean wounds and 17 involved clean-contaminated wounds. One study by the PREP-IT Investigators reported outcomes separately for closed and open fractures. The follow-up duration across the studies varied widely, ranging from 14 days to 3 years. The methodological quality assessment is presented in Figure 2, which illustrates the distribution of risk-of-bias judgments for all evaluated domains.

**Table 1:** Characteristics of RCT compare chlorhexidine-containing solutions with povidone-iodine solutions for skin disinfection to prevent SSIs.

Year	Study	Male sex (%)	Age	Sample Size	Type of surgical	Wound classification	Intervention	Control	Follow-up period
2005	Culligan et al. (33)	/	42.6/45	27/23	Hysterectomy	Clean-contaminated	4% chlorhexidine gluconate	10% povidone iodine	42d
2008	Veiga et al. (34)	/	>18	125/125	Plastic surgery	Clean	0.5% chlorhexidine	10% povidone-iodine	30d
2009	Paocharoen et al. (35)	63.6/55.2	50.5/56.2	250/250	Mixed	Clean/ clean-contaminated /contaminated	4% chlorhexidine in 70% isopropyl alcohol	Povidone iodine	30d
2010	Darouiche et al. (36)	58.9/55.9	53.3/52.9	409/440	Mixed	Clean-contaminated	2% chlorhexidine gluconate in 70% isopropyl alcohol	10% povidone–iodine	30d
2010	Sistla et al. (37)	99.0/96.5	/	200/200	Inguinal hernia repair	Clean	2.5% chlorhexidine with 70%	Povidone-iodine	30d
2013	Perek et al. (38)	73.3/84.4	62.2/70.2	45/46	Cardiac procedures	Clean	Chlorhexidine in 70% ethanol	Povidone-iodine in 50% propyl alcohol	30d
2013	Yeung et al. (39)	/	62.2/65.1	50/50	Genitourinary prosthetic surgery	Clean-contaminated	Chlorhexidine alcohol	Povidone-iodine	30d
2014	Abreu et al. (40)	/	/	24/32	Surgery for benign prostatic hyperplasia	Clean-contaminated	0.5% chlorhexidine in an alcohol base	0.5% povidone–iodine	3 years
2015	Srinivas et al. (41)	38/38	44.7/47.4	158/184	Upper abdominal	Clean-contaminated	0.5% chlorhexidine gluconate in 70% isopropyl alcohol	5% povidone–iodine	30d
2015	Bibi et al. (42)	37.5/40.4	40.4/41.31	168/220	Mixed	Clean/clean-contaminated	2% chlorhexidine gluconate in 70% alcohol	10% povidone-iodine	30d
2015	Kunkle et al. (43)	/	31/29.1	27/33	Cesarean delivery	Clean-contaminated	2% chlorhexidine gluconate with 70% isopropyl alcohol	10% povidone-iodine	14d
2015	Ngai et al. (44)	/	30.3/29.9	474/463	Cesarean delivery	Clean-contaminated	Chlorhexidine gluconate and alcohol	Povidone-iodine	30d
2016	Davies and Patel (45)	50/55	58/57	276/654	Cranial neurosurgery	Clean	Chlorhexidine gluconate	Povidone-iodine	30d
2016	Park et al. (46)	73.8/66.7	/	267/267	Gastrointestinal or hepatobiliary–pancreatic open surgery	Clean-contaminated	4% chlorhexidine gluconate	7.5% povidone–iodine	30d
2016	Salama et al. (47)	/	26.7/26.6	196/194	Cesarean sections	Clean-contaminated	Chlorhexidine and 70% alcohol group	Povidone-iodine and 70% alcohol group	30d
2016	Tuuli et al. (13)	/	28.3/28.4	572/575	Cesarean delivery.	Clean-contaminated	2% chlorhexidine gluconate with 70% isopropyl alcohol	8.3% povidone–iodine with 72.5% isopropyl alcohol	30d
2017	Broach et al. (48)	48.5/48.7	57.0/56.8	392/396	Colorectal surgery	Clean-contaminated	2% chlorhexidine gluconate and 70% isopropyl alcohol	Povidone–iodine and 74% isopropyl alcohol (0.7% available iodine solution)	30d
2017	Danasekaran et al. (49)	71.3/61.7	39.88/39.15	60/60	Elective abdominal surgeries	Clean-contaminated	2% chlorhexidine-alcohol	5% povidone-iodine	30d
2017	Patrick et al. (14)	45/51	49/41	203/204	Spinal surgery	Clean	2% chlorhexidine gluconate in 70% isopropyl alcohol	10% povidone-iodine available iodine in 95% alcohol,	30d
2017	Springel et al. (50)	/	28/28	461/471	Cesarean delivery	Clean-contaminated	2% chlorhexidine gluconate in 70% isopropyl alcohol	Povidone-iodine aqueous (0.75% available iodine solution)	30d
2019	Lakhi et al. (51)	/	32/49/32.61	524/590	Cesarean delivery.	Clean-contaminated	4% chlorhexidine gluconate solution	10% povidone-iodine	14d
2019	Peel et al. (52)	38.5/35.5	68/67	390/390	Hip or knee arthroplasty	Clean	0.5% chlorhexidine gluconate in 70% ethanol	10% povidone iodine in 70% alcohol (1% available iodine)	30d
2019	Saha et al. (53)	/	/	153/158	Cesarean delivery.	Clean-contaminated	Chlorhexidine–alcohol	Povidone-iodine	30d
2020	Dior et al. (54)	/	35.5/36.1	210/214	Gynecological Laparoscopic Surgery	Clean-contaminated	Chlorhexidine gluconate	Aqueous povidone-iodine solution	30d
2020	Gezer et al. (55)	/	51.9/54.4	55/55	Surgery for malignant or premalignant conditions of the uterus, cervix or ovary, or peritoneal carcinomatosis	Clean-contaminated	4% chlorhexidine gluconate with alcohol	10% povidone-iodine	30d
2020	Ritter et al. (56)	48.2/43.7	51.5/50.5	112/167	Lower limb trauma surgery	Clean	2% chlorhexidine and 70% isopropyl alcohol	1% PVP-I and 50% 2-propanol	180d
2021	Luwang et al. (57)	/	28.17/27.85	149/151	Cesarean delivery	Clean-contaminated	2% chlorhexidine–alcohol	10% povidone–iodine	30d
2022	Reid et al. (15)	61/56	69.17/68.25	141/493	Colorectal Surgery	Clean-contaminated	Chlorhexidine in 70% alcohol	Povidone-iodine in 70% alcohol	30d
2022	Smith et al. (16)	46/45	56/57	1076/1075	Mixed	Clean/clean-contaminated	0.5% chlorhexidine gluconate in 70% ethanol	10% povidone-iodine in 70% alcohol	30d
2024	Boisson et al. (17)	78.7/76.9	69/69	1621/1621	Major cardiac surgery via sternotomy	Clean	2% chlorhexidine in 70% isopropanol	5% povidone-iodine in 69% ethanol	90d
2024	The PREP-IT Investigators (18)	49.2/48.5	53.6/54.3	3425/3360	Closed-Fracture	Clean	2% chlorhexidine gluconate in 70% isopropyl alcohol	0.7% iodine povacrylex in 74% isopropyl alcohol	90d
		63.5/63.5	44.2/45	846/854	Open-Fracture	Contaminated			
2024	Widmer et al. (19)	67.1/66.3	65/65	1571/1750	Cardiac or abdominal surgery	Clean/ clean-contaminated /contaminated/infected	Chlorhexidine gluconate(20 mg chlorhexidine digluconate and 0.7 mL)	Povidone iodine(50.0 g propan-2-ol,1 g povidone iodine in 100 mL, resulting in 10% free available iodine)	30d

### Pooled surgical site infection rate

**Figure 3:**
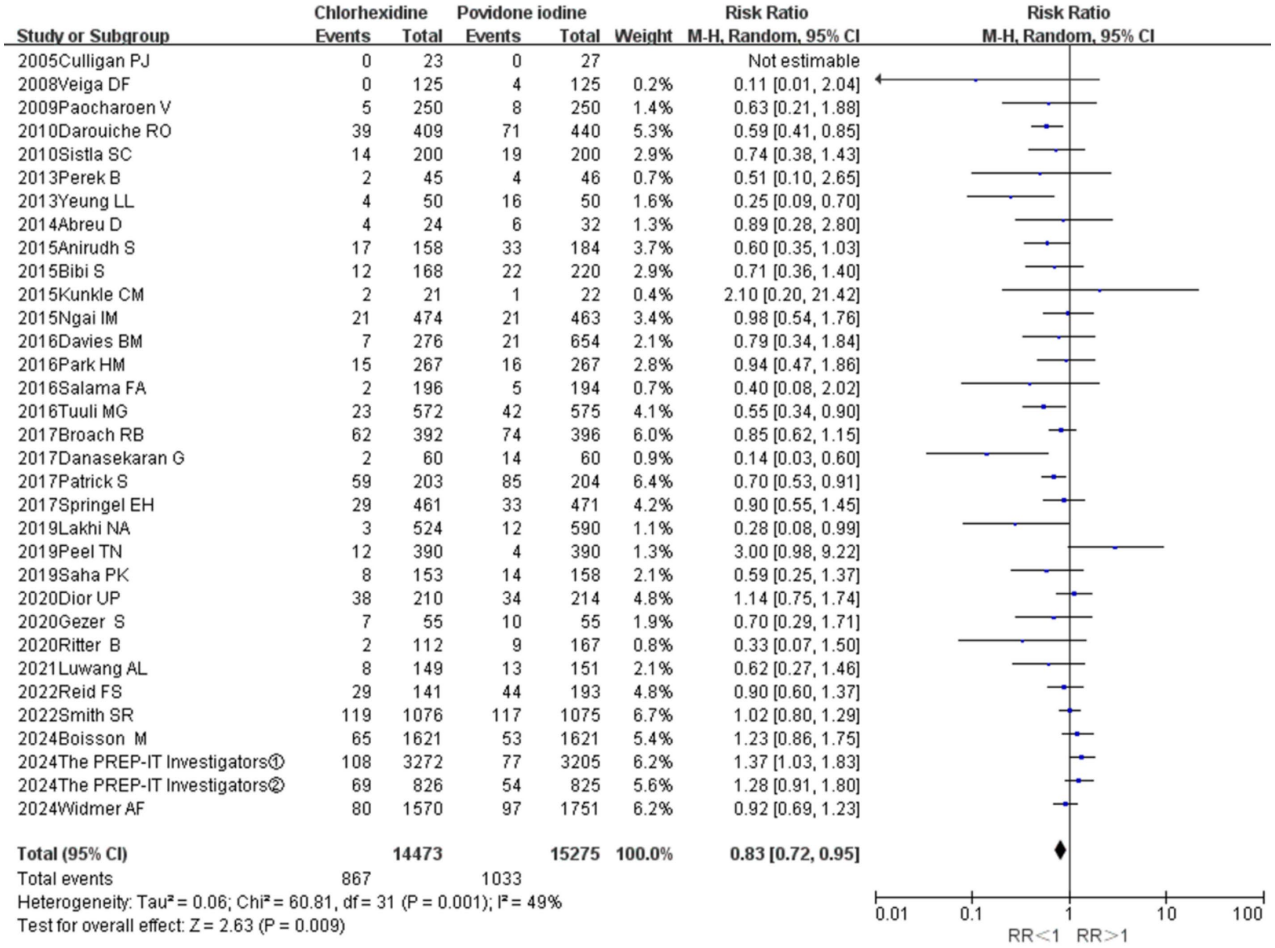
Meta-analysis forest plot: chlorhexidine compared with povidone-iodine solution for the prevention of surgical site infection.

**Figure 4:**
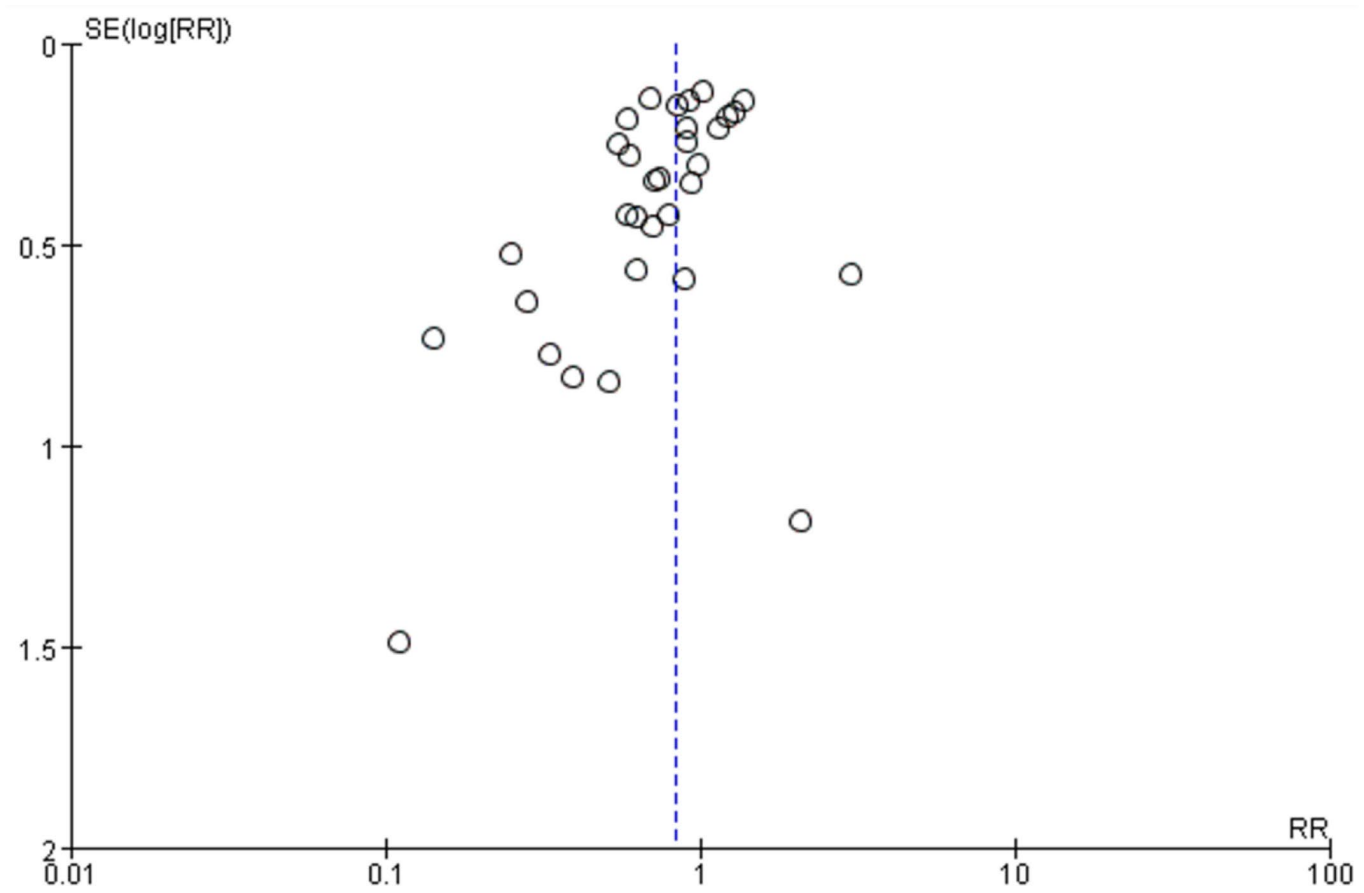
Meta-analysis funnel plot: chlorhexidine compared with povidone-iodine solution for the prevention of surgical site infection.

A total of 32 studies provided data comparing the incidence of surgical site infections (SSIs) between chlorhexidine (CHX) and povidone-iodine (PVI). Among the 29,748 patients included in the analysis, 1,900 (6.4%) developed an SSI. The incidence was lower in the CHX group (867/14, 473, 6.0%) compared to the PVI group (1,033/15, 275, 6.6%). The pooled random-effects meta-analysis demonstrated a superior effect of CHX over PVI in preventing SSIs (RR = 0.83, 95% CI 0.72–0.95, *p* = 0.009; Figure 3). Considerable heterogeneity was observed among the studies (*I*^2^ = 49%, *p* = 0.001). However, the funnel plot appeared symmetrical, indicating a low likelihood of publication bias (Figure 4). Furthermore, the leave-one-out sensitivity analysis confirmed the robustness of the pooled result.

### Wound classification

**Figure 5:**
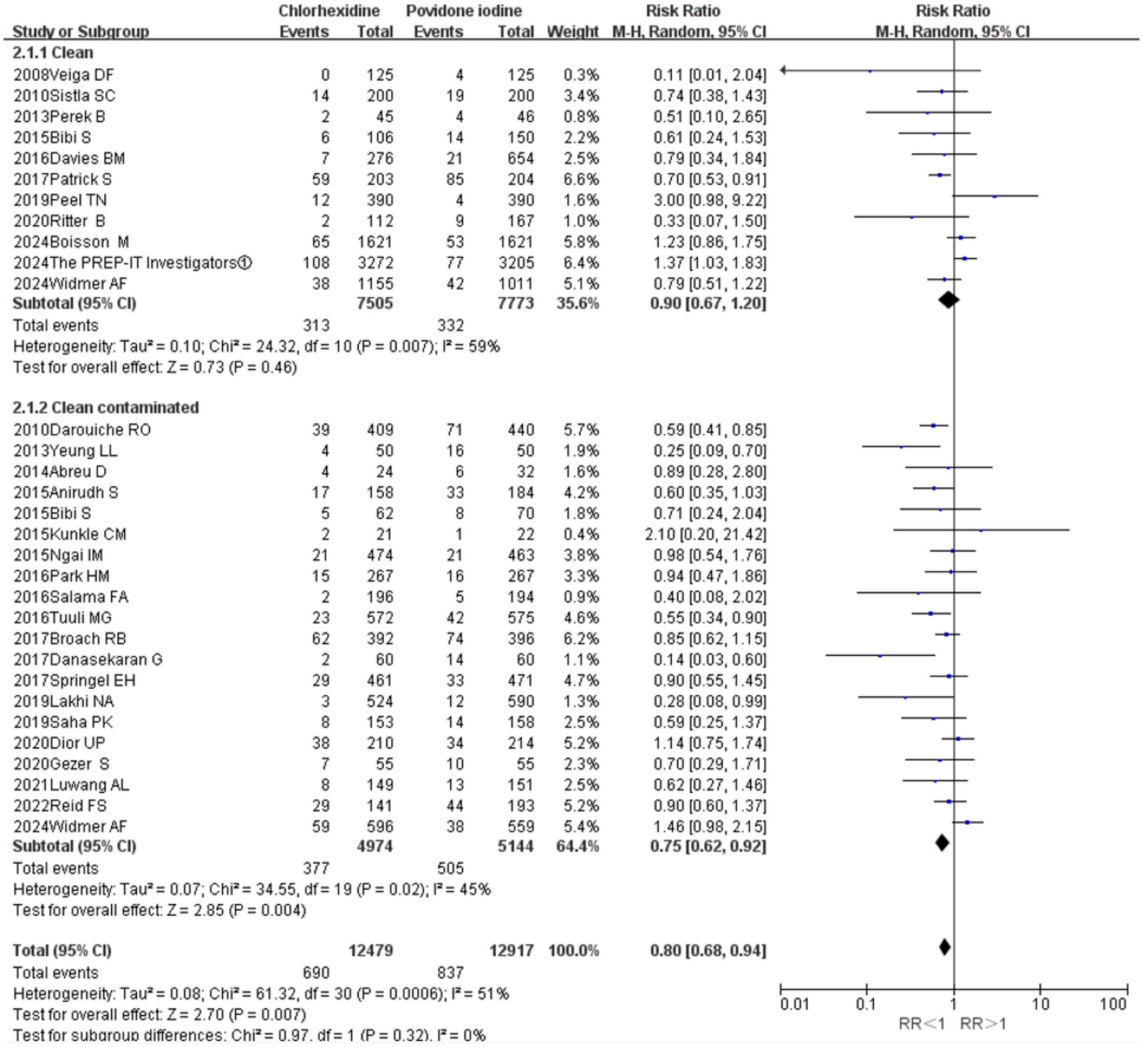
Forest plot of the subgroup analysis: clean surgery versus clean-contaminated surgery.

Stratified analysis by wound classification revealed that CHX was significantly superior to PVI in clean-contaminated surgeries (11 RCTs; RR = 0.75, 95% CI 0.62–0.92, *p* = 0.004; *I*^2^ = 45%; Figure 5). In contrast, no significant difference in SSI incidence was observed between the two antiseptics in clean surgeries (20 RCTs; RR = 0.90, 95% CI 0.67–1.20, *p* = 0.46; *I*^2^ = 59%; Figure 5).

### Classification of surgical site infections

**Figure 6:**
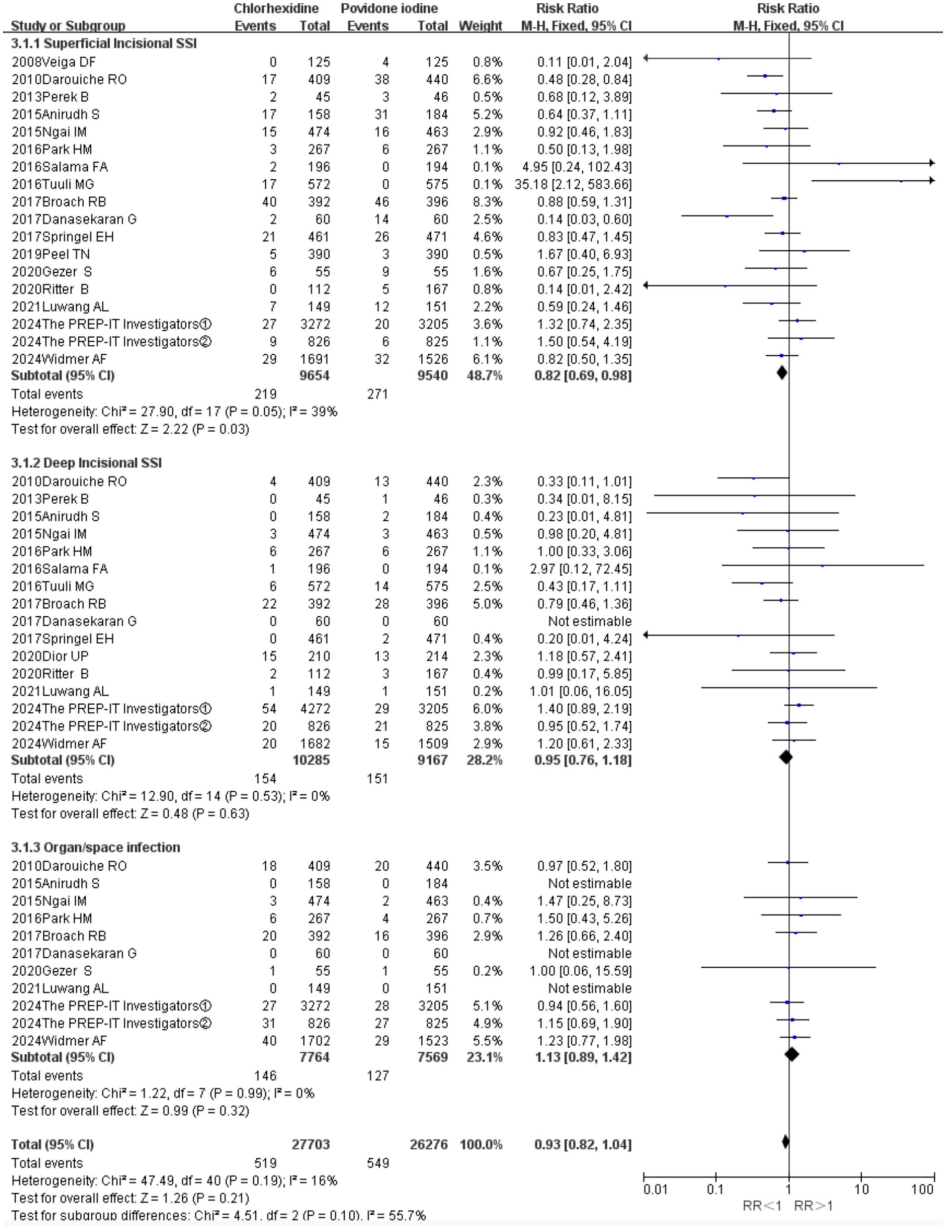
Forest plot of subgroup analysis: superficial incisional SSIs versus deep incisional SSIs versus organ-space SSIs.

Preventing superficial incisional SSIs (18 RCTs; RR = 0.82, 95% CI 0.69–0.98, *p* = 0.03; Figure 6). In contrast, no significant differences were observed between the two antiseptics for the prevention of deep incisional SSIs (16 RCTs; RR = 0.95, 95% CI 0.76–1.18, *p* = 0.63) or organ-space SSIs (11 RCTs; RR = 1.13, 95% CI 0.89–1.42, *p* = 0.32; Figure 6). The analyses for deep incisional (*I*^2^ = 0%) and organ-space SSIs (*I*^2^ = 16%) showed negligible heterogeneity, while the analysis for superficial SSIs indicated low to moderate heterogeneity (*I*^2^ = 39%).

### Diverse formulations

**Figure 7:**
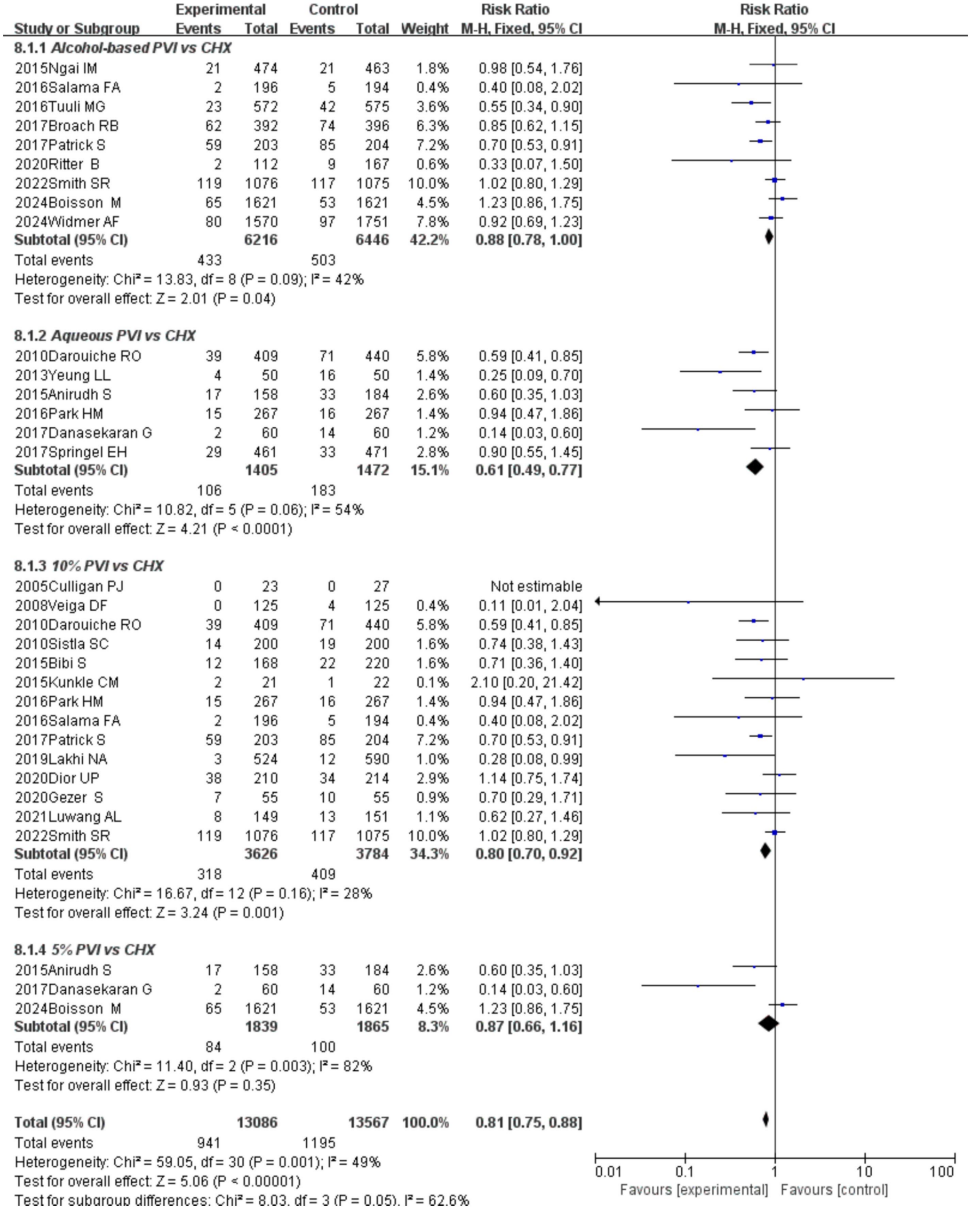
Forest plot of subgroup analysis: diverse formulations.

The meta-analysis demonstrated a superior effect of CHX over alcohol-based PVI (RR = 0.88, 95% CI 0.78–1.00, *p* = 0.040; Figure 7) and aqueous PVI (RR = 0.61, 95% CI 0.49–0.77, *p* = 0.001) in preventing SSIs. Meta-analysis of 14 RCTs revealed CHX was associated with a reduced risk of preventing SSIs when compared to 10% PVI (RR = 0.80, 95% CI 0.70–0.92, *p* = 0.001). In contrast, no significant differences were observed between CHX and 5% PVI for the prevention of deep incisional SSIs.

### Type of surgical

The fixed-effects pooled analysis revealed that CHX-containing solution was superior to PVI in the prevention of postoperative surgical site infection in cesarean delivery (eight RCTs, RR = 0.69, 95% CI 0.54–0.89, *p* = 0.004; Supplementary Figure 1). Potential heterogeneity was not observed in the included studies (*I*^2^ = 0%, *p* = 0.46). There were no significant differences in rates of SSIs between chlorhexidine and PVI in the prevention of gastrointestinal surgeries (three RCTs, RR = 0.87, 95% CI 0.69–1.11, *p* = 0.26), orthopedic surgery (five RCTs, RR = 1.09, 95% CI 0.93–1.29, *p* = 0.29), and cardiac procedures (three RCTs, RR = 1.02, 95% CI 0.82–1.27, *p* = 0.87).

### Bacterial decolonization and fever episodes

Meta-analysis of 11 RCTs revealed chlorhexidine was associated with a reduced risk of bacterial decolonization when compared to PI (RR = 0.38, 95% CI 0.26–0.57, *p* = 0.001; Supplementary Figure 2). Furthermore, there were also statistical differences between chlorhexidine and PVI in the incidence of fever episodes (RR = 0.57, 95% CI 0.35–0.92, *p* = 0.02; Supplementary Figure 3).

### Trial sequential analysis

**Figure 8:**
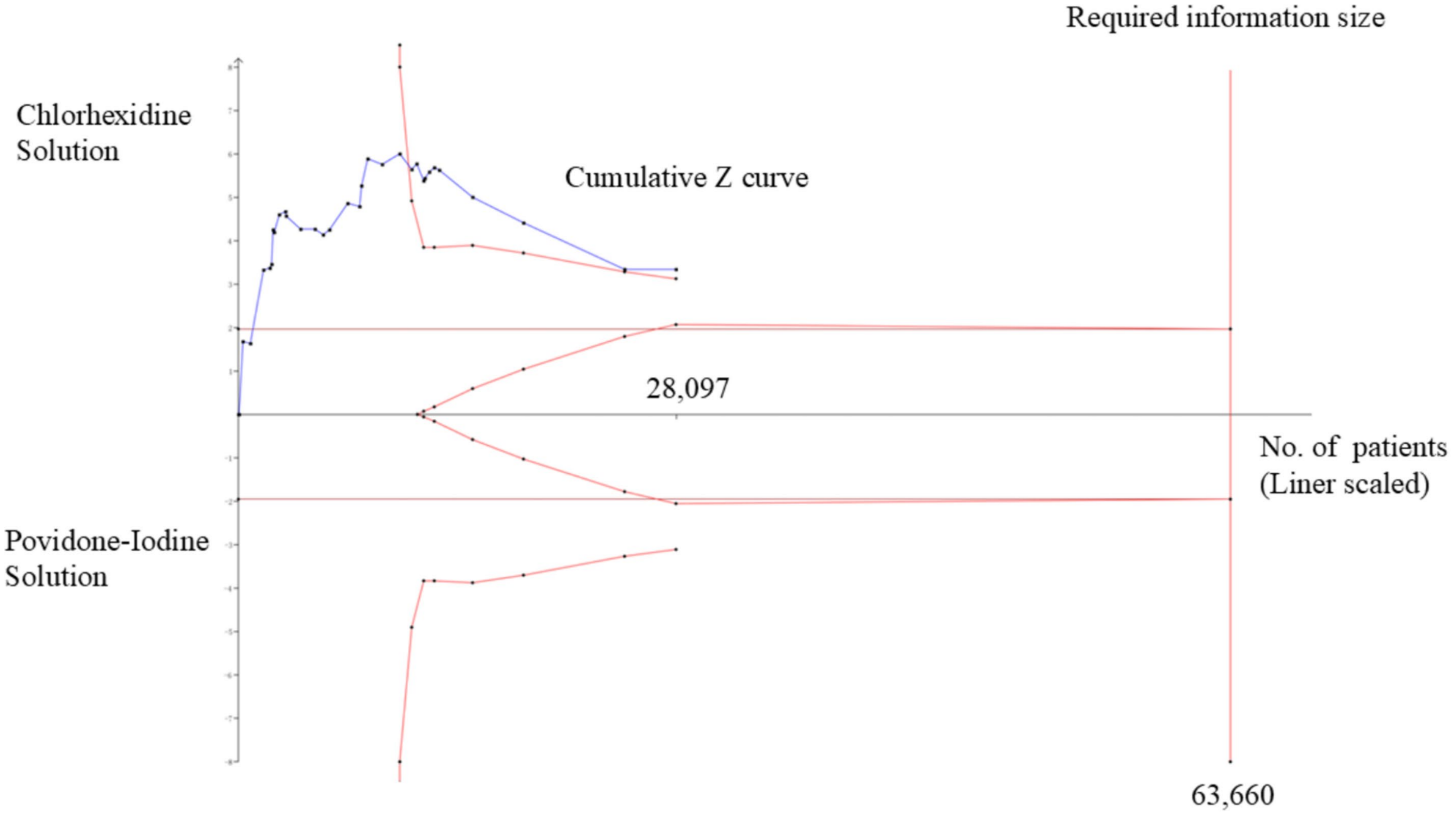
Trial sequential analysis (TSA) of pooled surgical site infections (SSIs) rate. Heterogeneity-adjusted required information size of 63,660 participants, calculated with a two-sided conventional boundary with a 5% type I error rate and an 80% statistical power.

TSA showed that 44.14% (28,097 out of 63,660 patients) of the heterogeneity-adjusted information size required was accrued. We also found that the cumulative Z-curve crossed the trial sequential monitoring boundary, providing robust evidence of chlorhexidine compared to povidone-iodine solution for the prevention of surgical site infection based on the sample size (Figure 8).

## Discussion

Preoperative skin antisepsis with chlorhexidine was associated with a lower risk of surgical site infections (SSIs) overall compared to povidone-iodine. This beneficial effect was primarily driven by a significant reduction in SSI risk following clean-contaminated surgeries and in the incidence of superficial incisional SSIs. In contrast, no significant differences between the antiseptics were observed for the prevention of deep incisional or organ-space SSIs.

This meta-analysis confirms that chlorhexidine (CHX) is superior to povidone-iodine (PVI) in preventing overall surgical site infection (SSIs), thereby reinforcing the findings of a prior meta-analysis by Chen et al. (2020) (7). Notably, the present review strengthens the existing evidence by exclusively including randomized controlled trials (RCTs), whereas the earlier work incorporated observational designs that may introduce bias. Furthermore, our study provides novel insights by demonstrating that the superiority of CHX is specifically significant in preventing superficial incisional SSIs and in clean-contaminated surgeries—associations not previously detailed in the literature. Our findings align with the conclusions of Privitera et al. (8), which offered moderate-quality evidence supporting CHX. While that analysis was limited by the number of available RCTs, our updated synthesis includes a substantially larger body of evidence from high-quality RCTs. Contrary to these conclusions, a 2022 meta-analysis from the National Institute of Health Research Unit on Global Surgery found no significant differences between alcoholic CHX and aqueous PVI (13–16). This discrepancy may be partly explained by their comparison not including alcoholic PVI formulations, potentially influencing the pooled effect. Regarding safety, although our review did not perform a quantitative analysis of adverse skin reactions due to inconsistent reporting definitions, the existing literature cited in these prior reviews suggests that CHX is generally well-tolerated with a low incidence of hypersensitivity (8).

Several high-quality RCTs have been reported in the last 2 years. An RCT published in Intensive Care Med (17) found skin disinfection at the surgical site using CHX-alcohol was not superior to PVI-alcohol in reducing surgical site infection rates among patients requiring sternotomy for major heart or aortic surgery. This finding differs from previously published results, and the sample size was relatively large. Two other RCTs published in NEJM (18) and JAMA (19) were also included in this meta-analysis. A previous study reported the results in patients with closed extremities and open fractures. The latest two studies in 2024 and 2025 (20) were excluded from our meta-analysis because of the lack of SSI rates and study design (a prospective observational analysis).

Povidone-iodine is a microbicide skin preparation with broad-spectrum antiseptic properties and local tolerability, which can rapidly penetrate microorganisms and attack their nucleotides, fatty acids, and thiol groups, while inhibiting microbial protein synthesis by oxidizing thiol groups (21). In clean surgery, we found no difference between CHX and PVI. CHX is a cationic chlorinated biguanide that precipitates in the bacterial cell membranes and cytoplasmic components. CHX resists neutralization by organic materials, is active over a wider pH (5–8) than iodine compounds, has a more prolonged bactericidal action, and has a lesser incidence of skin sensitization (22, 23). Macias JH (24) demonstrated a longer reduction of colony-forming units by cell-bound CHX. Furthermore, CHX remains activated in the presence of organic fluids, such as blood or pus, in contrast to iodophores, which become inactivated (25). Our study proved that the advantage of CHX over PVI is obvious in the prevention of superficial incisional SSIs. Chlorhexidine-alcohol is a newer skin preparation agent commonly composed of 2% chlorhexidine gluconate and 70% isopropyl alcohol (26). Although it is more expensive than PVI, it is reported to have a more rapid onset of action than PI and persistent activity in the presence of body fluids. Literature on the most appropriate concentration of chlorhexidine is sparse. In a randomized trial of 100 patients, Casey compared 2% chlorhexidine with 0.5% chlorhexidine. CHX 2% significantly reduced the number of microorganisms on the skin but did not reduce the incidence of SSIs (27).

However, the study outcomes of CHX-alcohol combinations are often attributed to CHX alone. The rate of incorrect attribution among the articles that we assessed ranged from 29% for catheters to 43% for surgery (28). Alcohol, which is a conventional antiseptic, may play a critical role in this process. WHO guidelines recommend the use of chlorhexidine alcohol-based antiseptic solutions for surgical site skin preparation (3). A recent meta-analysis found good evidence favoring CHG-alcohol (C-Alc) combinations over aqueous PVI, the most commonly tested alternative, in all three areas of skin antisepsis (28). Randomized controlled trials involving alcoholic and aqueous chlorhexidine are required. Given the increased risk and the hypothesis that the improvement in SSI rate in other trials is due to the addition of alcohol, a three-armed RCT compared povidone-iodine in an alcohol base (PI-Alc) to povidone-iodine in an aqueous base (PI-Aq) alongside a non-inferiority trial and found PI-Alc to be non-inferior to C-Alc and not superior to PI-Aq. Furthermore, the risks of surgical fire, chemical burns, anaphylaxis, and cost should be considered when choosing an appropriate skin preparation (15). However, considering the limited data, we could not perform a subgroup analysis. Furthermore, SSI prevention is a multi-factorial endeavor. While skin antisepsis plays an important role, other evidence-based measures—such as appropriate antibiotic prophylaxis, glycemic control, maintenance of normothermia, and adherence to sterile technique—are also critical components within comprehensive SSI prevention bundles.

The subgroup analysis in this study revealed a critical and nuanced finding: the relative efficacy of antiseptics is highly dependent on the formulation of PVP-I. While the overall analysis suggested that CHX might be superior to PVP-I, the picture became more complex when disaggregated into carrier solution. First, consistent with most previous meta-analyses, this study found that CHX was significantly superior to aqueous PVP-I in preventing surgical site infections (29). This is likely attributable to the excellent residual activity of CHX, which provides prolonged antimicrobial effects. In contrast, the iodine activity of aqueous PVP-I gradually diminishes after drying and is more easily inactivated by organic matter such as blood or serous secretions (30, 31). Alcohol-based PVP-I, however, combines the rapid bactericidal capacity of alcohol with the broad-spectrum antimicrobial activity and sustained action of iodine. The alcohol carrier not only rapidly kills microorganisms itself but also enhances the penetration of iodine compounds into deeper skin structures (32). This combination of rapid initial kill and persistent residual antimicrobial action may render alcohol-based PVP-I a highly powerful antiseptic option in specific clinical scenarios.

Several limitations inherent in this meta-analysis warrant acknowledgment. First, the pooled results are derived from a heterogeneous mix of surgical procedures (e.g., plastic, spinal, and prosthetic surgeries), and the effect of CHX may differ among them. Our ability to perform subgroup analyses was constrained by a lack of reporting on other prevalent surgery types. Second, variations in CHX concentration, application technique, and contact duration across studies could have influenced the observed effect on SSI prevention. Furthermore, our analysis primarily contrasted the active agents (CHX vs. PVI) and could not fully disentangle the confounding effect of different solvents (alcohol vs. aqueous), potentially reducing the reliability of the findings. The exclusion of non-English studies may have introduced language and publication biases. Our analysis was also limited to clean and clean-contaminated wounds due to the absence of data on more contaminated wound classes. Moreover, the exclusion of pediatric populations means that the results are not generalizable to patients under 18 years of age. Finally, the highly variable SSI monitoring periods (ranging from 14 days to 3 years) represent a major source of bias, potentially leading to an underestimation of infection risk and hindering direct comparisons. Although a sensitivity analysis suggested robustness, this bias cannot be fully eliminated, urging caution in interpreting the summary results.

## Conclusion

This meta-analysis demonstrated that CHX-containing solutions were more effective than PVI-containing solutions in preventing postoperative SSIs, particularly in clean-contaminated surgeries. As for the classification of SSIs, the advantage of CHX versus PVI is obvious in preventing superficial incisional SSIs, but not in deep incisional SSIs and organ-space SSIs. SSI prevention is a multi-factorial endeavor. While skin antisepsis plays an important role, other evidence-based measures—such as appropriate antibiotic prophylaxis, glycemic control, maintenance of normothermia, and adherence to sterile technique—are also critical components within comprehensive SSI prevention bundles.

## Data Availability

The original contributions presented in the study are included in the article/Supplementary material, further inquiries can be directed to the corresponding author/s.

## Footnotes

^1^ http://www.Ctu.dk.tsa
