# Supplementary figures and images for: Chlorhexidine versus povidone-iodine for surgical site infection prevention: an updated meta-analysis and trial sequential analysis of randomized controlled trials
Source: Front Med (Lausanne). 2025 Nov 20;12:1641815. doi: 10.3389/fmed.2025.1641815 (PMC12675439; doi:10.3389/fmed.2025.1641815)

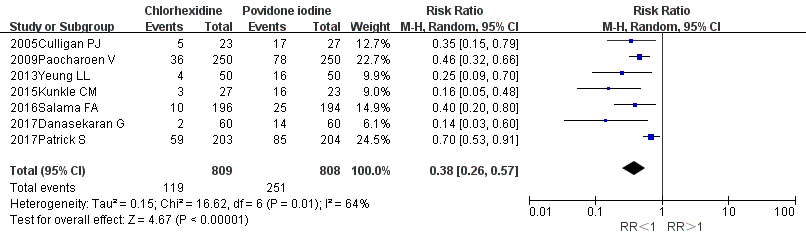

Supplement: Supplementary file 1 [file Image_1.png]

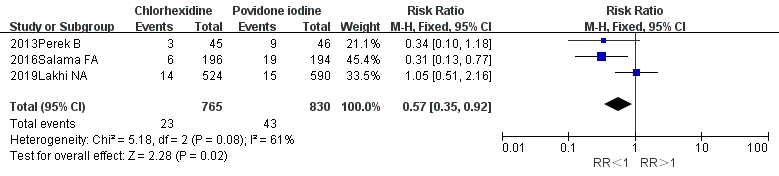

Supplement: Supplementary file 2 [file Image_2.png]

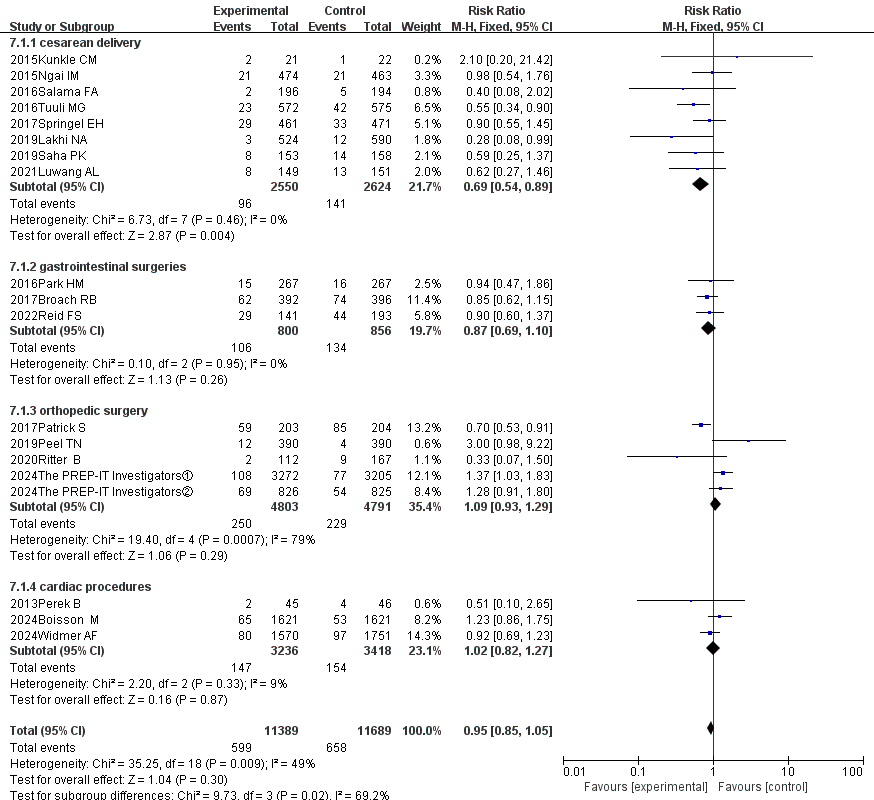

Supplement: Supplementary file 3 [file Image_3.png]
